# Anti-diabetic retinopathy molecular mechanism of Dihuang Yinzi: insights from network pharmacology, metabolomics, and microbiome analysis

**DOI:** 10.3389/fmed.2026.1793936

**Published:** 2026-05-15

**Authors:** Jinmiao Chai, Wanwei Gui, Junlong Zhang, Wenbin He, Qinqing Li

**Affiliations:** 1 Shanxi University of Chinese Medicine, Jinzhong, Shanxi, China; 2 Shanxi Provincial Key Laboratory of TCM Encephalopathy, Jinzhong, Shanxi, China; 3 National International Joint Research Center for Molecular Traditional Chinese Medicine, Jinzhong, Shanxi, China

**Keywords:** diabetic retinopathy, Dihuang Yinzi, key target genes, metabolomic, microbiome

## Abstract

**Background:**

Diabetic retinopathy (DR) represents a major microvascular complication arising from diabetes mellitus, characterized by multifactorial pathogenesis encompassing genetic, metabolic, and microbial components. While Dihuang Yinzi (DHYZ), a traditional Chinese herbal formulation, exhibits therapeutic promise for DR management, the precise mechanistic underpinnings warrant further investigation. This research sought to elucidate critical target genes, metabolic compounds, and microbial species implicated in DHYZ's therapeutic action against DR.

**Methods:**

We constructed a murine DR model and procured biological specimens from 18 animals distributed across three experimental cohorts (control, disease model, and DHYZ-treated groups, *n* = 6 per group) for metabolomic profiling and microbiome characterization. Candidate genes emerged from overlapping DHYZ-associated targets with DR-linked genes. Through six computational algorithms within the cytoHubba plugin, we pinpointed pivotal target genes. Molecular docking studies examined binding affinity between essential target proteins and bioactive constituents. Metabolomic and microbiome datasets underwent differential expression analysis to enumerate candidate metabolites and microbial taxa, respectively. Finally, Spearman correlation-based integrative omics analysis distinguished critical metabolites and key microbial species.

**Results:**

We identified 110 candidate genes and five key target genes (STAT3, IL6, TNF, ESR1, and IL1B). Molecular docking analysis revealed strong binding interactions between ESR1 and six corresponding active compounds, with the highest binding affinity observed for naringenin. Additionally, metabolomic analysis identified 50 candidate metabolites, and microbiome analysis revealed 24 candidate microbes. Spearman correlation analysis further pinpointed 30 key metabolites and 18 key microbes.

**Conclusion:**

This study elucidates five key target genes, 30 key metabolites, and 18 key microbes through which DHYZ may exert its therapeutic effects in DR. These findings provide valuable insights and a foundational reference for understanding the multi-omics mechanism of DHYZ in the treatment of diabetic retinopathy.

## 1 Introduction

Diabetic retinopathy (DR), a major microvascular complication of diabetes, is the predominant cause of vision loss in the global working-age population. Epidemiological studies estimate that DR affects nearly one-third of individuals with diabetes, corresponding to more than 140 million people worldwide. The risk of developing DR exceeds 60% in those with diabetes duration longer than 10 years (1, 2). According to the latest demographic statistics, approximately 103.12 million people with diabetes were affected by DR worldwide in 2020. This number is projected to increase to 129.84 million by 2030 and 160.50 million by 2045, indicating that more than 57 million new DR cases will be added globally over the next two decades (3, 4). As one of the countries with the heaviest diabetes burden worldwide, China exhibits a particularly prominent epidemiological profile of DR. A study based on the Global Burden of Disease (GBD) 2021 data predicts that the disease burden of DR in China will continue to rise over the next 15 years, which is closely associated with the large population of patients with diabetes and the rapidly aging social structure in China (5).

A substantial diagnosis gap for DR exists globally, with more than 50% of DR cases remaining undiagnosed; this proportion is even higher in developing countries with limited medical resources, such as India (6). Such undiagnosed conditions expose numerous patients to the risk of irreversible visual impairment without any obvious subjective symptoms. Despite advances in ophthalmology, current management of DR faces significant challenges, including inadequate early screening and the limited efficacy of single-target therapeutic interventions, which often result in high recurrence rates. The pathophysiology of DR is multifactorial, involving chronic hyperglycemia-induced endothelial damage in retinal vessels (7), pericyte loss, and disruption of the blood-retinal barrier (8, 9). Consequently, a comprehensive understanding of DR is crucial for developing effective and targeted therapeutic strategies, which could facilitate early detection and improve clinical outcomes for DR patients (10, 11). In summary, the global prevalence of DR continues to expand, with a persistently high proportion of undiagnosed cases and numerous challenges in clinical management, leading to a steadily increasing disease burden. Meanwhile, against the backdrop of uneven distribution of medical resources, the gaps in the prevalence, diagnosis, and treatment of DR across regions and populations have further widened, making it a major global public health issue that urgently requires concerted international attention and response.

To address the complexity of DR pathogenesis, network pharmacology has emerged as a valuable interdisciplinary approach that integrates principles and analytical methods from systems biology and network science (12). It examines complex disease and drug action networks from a systems-level perspective to investigate multi-target pharmacological effects (13, 14). By modeling these intricate interactions, it facilitates the comparative analysis of pathological and drug-response systems, enabling the identification of critical disease drivers and therapeutic targets. Key advantages of this approach include a more comprehensive understanding of drug mechanisms, promotion of novel drug development, support for personalized treatment design, enhancement of treatment efficacy and safety, and efficient discovery of drug targets and modes of action. As a result, network pharmacology provides more accurate and reliable guidance for both drug discovery and clinical translation (15). Given the multi-component and multi-target characteristics of traditional Chinese medicine, network pharmacology represents an ideal approach to systematically investigate the therapeutic mechanisms of herbal formulae such as DHYZ in the treatment of DR.

Dihuang Yinzi (DHYZ) is a traditional Chinese medicinal formula consisting of 15 herbs, with a history of use in the treatment of neurodegenerative diseases such as Alzheimer's disease. The pharmacological actions of its constituent herbs are as follows: *Rehmannia glutinosa* and *Cornus officinalis* are known to tonify the kidney and replenish essence; *Aconitum carmichaelii, Cinnamomum cassia, Morinda officinalis*, and *Cistanche deserticola* function to warm and invigorate kidney yang; *Ophiopogon japonicus, Dendrobium nobile*, and *Schisandra chinensis* work to nourish yin and astringe bodily fluids; *Acorus tatarinowii* and *Polygala tenuifolia* help resolve phlegm and open orifices, thereby clearing turbid phlegm from visual pathways; *Poria cocos* promotes diuresis, eliminates dampness, strengthens the spleen, and harmonizes the stomach; *Mentha haplocalyx* exhibits antibacterial and anti-inflammatory properties; and *Zingiber officinale* and *Ziziphus jujuba* serve to harmonize the stomach, tonify the middle energizer, and regulate gastric qi. Together, these components act synergistically to enhance kidney essence, resolve phlegm, and improve visual function (16, 17). Elucidating the biological mechanisms of DHYZ in DR is of great importance, as it may uncover novel therapeutic targets and pave the way for innovative treatment strategies.

Multi-omics integrated analysis combines biological data from multiple levels—such as metabolomics and microbiomes—enabling researchers to observe gene functions and regulatory mechanisms from diverse perspectives and to elucidate complex molecular interactions and signaling pathways. By leveraging complementary omics datasets, this approach enables in-depth exploration of biological processes, integrating data from phenotypic manifestations to molecular mechanisms, thereby providing novel insights into fundamental biological mechanisms and disease pathogenesis. Applying multi-omics integrated analysis to uncover the complex pathogenesis of DR holds promise for providing critical new insights into its molecular underpinnings and facilitating the development of targeted therapeutic strategies (18).

This study employed network pharmacology to identify key targets of DHYZ in the intervention of DR. Using metabolomic and microbiome sequencing data from control, model, and DHYZ-treated groups, we conducted integrated bioinformatic analyses to identify candidate metabolites and microbes linked to the therapeutic mechanisms of DHYZ across omics layers. Subsequent multi-omics integration highlighted critical metabolites and key microbial taxa, offering novel theoretical and empirical support for future clinical research and treatment development in DR.

## 2 Materials and methods

### 2.1 Animals

Eighteen male db/db mice at 9 weeks of age, SPF-grade, with mean body weight of 40.88 ± 2.15 g, underwent random allocation into model and DHYZ treatment cohorts via random number table methodology. An additional nine age-matched male db/m mice (mean body weight: 28.39 ± 1.39 g) constituted the control cohort. All experimental subjects were procured from Changzhou Cavens Laboratory Animal Co., Ltd. [License No.: SCXK (Jiangsu) 2021-0013] and housed under specific pathogen-free (SPF) standards at the Animal Experiment Center of Shanxi University of Chinese Medicine. Environmental conditions were maintained at 23 ± 1 °C with 50%−65% relative humidity throughout the study period. This experimental protocol received ethical approval from the Animal Ethics Committee of Shanxi University of Chinese Medicine (Approval No.: 2021DW238).

Following anesthesia via isoflurane inhalation, three mice from each group underwent cardiac perfusion. Perfusion was continued until the liver turned pale, indicating complete fixation. Eyeballs were then enucleated and fixed in 4% paraformaldehyde (PFA) for 24 h. The remaining six mice per group were used for serum and fecal sample collection. These samples were subsequently analyzed using metabolomic profiling and 16S rRNA gene sequencing of the gut microbiota. Corresponding sample identifiers for both metabolomics and Microbiome sequencing are provided in the Supplementary Table.

### 2.2 Preparation for DHYZ

The corresponding sample identifiers are provided in the Supplementary Table. All herbs were supplied by Beijing Tongrentang Co., Ltd. The mixture was soaked in water for 30 min, decocted twice for 30 min each time, filtered, and then concentrated to a final concentration of 2 g/ml (crude drug). The equivalent dose for mice was calculated based on body surface area conversion. The adult human daily dose of DHYZ is 99 g, corresponding to a dose of 30.03 g/kg for mice in the treatment group. The control and model groups received an equal volume of normal saline. All treatments were administered continuously for 28 days. After anesthesia and euthanasia, serum and colonic content samples were collected from six mice per group. These samples were subjected to metabolomic profiling and 16S rRNA gene sequencing of the gut microbiota.

### 2.3 Retinal morphological observation

Following fixation, retinal tissues were dehydrated, cleared, and embedded in paraffin. Sections were then sequentially stained with hematoxylin to visualize nuclei, differentiated in hydrochloric acid alcohol, blued, counterstained with eosin for cytoplasmic visualization, dehydrated through a graded ethanol series, cleared in xylene, and finally mounted with neutral balsam. Stained retinal sections from each group were scanned using a digital slide scanner, and pathological alterations were examined and documented.

### 2.4 Prediction of target genes for DHYZ

DHYZ is a formula comprising 15 herbs (Table 1). To identify its active compounds, the TCMSP database (https://tcmsp-e.com/tcmsp.php) was first searched by entering each of the 12 herb names individually in the “Herbname” field, excluding *Dendrobium officinale* Kimura & Migo (Shi Hu), *Ophiopogon japonicus(L.f)* Ker-Gawl. (Mai Dong), and *Polygala tenuifolia* Willd. (Yuan Zhi). The active compounds of these 12 herbs were then mapped to their target genes using the UniProt database (19) (https://www.uniprot.org/). After merging and deduplication, these targets were designated as target gene 1.

**Table 1:** Component herbs of DHYZ.

Botanical plant name	Chinese name
*Rehmannia glutinosa* Libosch.	Di Huang
*Morinda officinalis* How	Ba Ji Tian
*Cornus officinalis* Siebold.et Zucc	Shan Zhu Yu
*Dendrobium nobile* Lindl.	Shi Hu
*Cistanche deserticola* Y.C.Ma	Rong Cong Rong
*Aconitum carmichaelii* Debx.	Fu Zi
*Schisandra chinensis* (Turcz.) Baill.	Wu Wei Zi
*Cinnamomum cassia* Presl	Rou Gui
*Poria cocos* (Schw.) Wolf	Fu Ling
*Ophiopogon japonicus* (L.f) Ker-Gawl.	Mai Dong
*Acorus calamus* L.	Chang Pu
*Polygala tenuifolia* Willd.	Yuan Zhi
*Mentha haplocalyx* Briq.	Bo He
*Zingiber officinale* Rosc.	Sheng Jiang
*Ziziphus jujuba* Mill.	Da Zao

Next, the active compounds and corresponding target genes of the remaining three herbs—Shi Hu, Mai Dong, and Yuan Zhi—were retrieved from the HERB database (20) (http://herb.ac.cn). These targets were merged and deduplicated to form target gene 2.

Finally, target set 1 and target gene 2 were combined and deduplicated to obtain the complete set of predicted target genes for DHYZ. To ensure data reliability, TCMSP and HERB databases were used in a complementary manner. TCMSP served as the primary source for the 12 herbs, while HERB supplemented the three herbs not fully covered by TCMSP. The ETCM database was not included as it lacks complete information for key herbs such as Rehmannia glutinosa. The combined use of TCMSP and HERB thus provided comprehensive coverage of active compounds and targets for all 15 herbs in DHYZ.

### 2.5 Identification and enrichment analysis of candidate genes

DR-associated target genes were retrieved from Genecards (https://www.genecards.org/), OMIM (https://www.omim.org/), and Therapeutic Target Database (21) (https://db.idrblab.net/ttd/) employing “diabetic retinopathy” as the query. Targets from OMIM and TTD were merged with the top 500 relevance-ranked genes from Genecards. Following consolidation and deduplication, we established the DR-associated target gene set. Candidate genes were determined by intersecting DHYZ-predicted targets with DR-associated genes via the VennDiagram package (v 1.7.3) (22).

Gene Ontology (GO) and Kyoto Encyclopedia of Genes and Genomes (KEGG) pathway enrichment analyses were conducted using clusterProfiler package (v 4.2.2) (23) to elucidate biological functions and pathways of candidate genes, applying *P* < 0.05 as the significance cutoff. Results underwent *p*-value-based ascending sorting. For each GO domain-biological process (BP), cellular component (CC), and molecular function (MF)-the five most significantly enriched terms were chosen, alongside the ten leading KEGG pathways, for subsequent analysis and graphical representation.

### 2.6 Identification of key target genes

Subsequently, candidate genes were imported into the STRING database (24) (http://string-db.org) for protein-protein interaction (PPI) network construction using a confidence threshold exceeding 0.90. Cytoscape software (v 3.9.1) (25) visualized interactions among the top 15 candidates prioritized by the Closeness algorithm. The cytoHubba plugin facilitated additional refinement by assessing genes across six topological parameters: Closeness, Degree, Edge Percolated Component (EPC), Maximal Clique Centrality (MCC), Maximum Neighborhood Component (MNC), and Radiality. From each ranking approach, the top 10 genes were extracted, with overlapping genes designated as key targets via the UpSetR package (v 1.4.0) (26). Finally, Cytoscape integrated and visualized key targets, their cognate bioactive compounds, and source herbs as a comprehensive network.

### 2.7 Molecular docking analysis

Molecular docking was employed to examine binding interactions between pivotal target proteins and their cognate bioactive compounds. Three-dimensional compound structures were obtained from PubChem database (https://pubchem.ncbi.nlm.nih.gov/), while protein architectures were sourced from UniProt database. CB-Dock web server (27) (https://cadd.labshare.cn/cb-dock/php/blinddock.php) executed docking simulations. Binding energy calculations quantified ligand-target interaction strength, wherein lower energies signify enhanced binding affinity, with values below −5 kcal/mol indicating favorable interactions. PyMOL software (v 2.5) rendered visualization of docking outcomes (28).

### 2.8 Metabolome sequencing and data pre-processing

The collected samples were thawed on ice, and metabolites were extracted using pre-chilled 50% methanol. After vortexing and incubation at room temperature, the samples were stored at −80 °C. The extracts were then centrifuged, and the supernatant was transferred to a new 96-well plate and stored at −80 °C until LC-MS analysis. To ensure data quality, quality control (QC) samples were included in each batch.

Chromatographic separation was performed using an ultra-high-performance liquid chromatography (UPLC) system equipped with a dedicated column under a specific gradient elution program. Mass spectrometry analysis was carried out on a high-resolution TripleTOF 5,600 plus mass spectrometer, which alternated between positive and negative ion modes. Data were acquired in information-dependent acquisition (IDA) mode with standard calibration. System stability was monitored by injecting QC samples throughout the acquisition process.

The raw data were converted to mzXML format using Proteowizard (v 3.0) (29). Peak detection, alignment, and integration were subsequently performed using XCMS (30). For initial metabolite identification, the accurate m/z values were queried against the Human Metabolome Database (HMDB) and KEGG database using MetaX software (v 2.0.0) (31). For further annotation, secondary spectra generated from ion fragmentation were matched against a curated spectral library.

### 2.9 Principal component analysis (PCA) and orthogonal partial least squares discriminant analysis (OPLS-DA)

Following this, PCA was employed using the stats (v 4.3.1) package to assess the quality of metabolome data among the DHYZ, model, and control groups. To illustrate the relationship between metabolite expression levels and sample categories, while maximizing sample separation and predicting sample categories, OPLS-DA was applied to the metabolome sequencing data for the model and control groups, as well as for the DHYZ and model groups, in both positive and negative ion modes using the ropls (v 1.38.0) package. The Variable Importance in Projection (VIP) values for each metabolite were then obtained. Additionally, multiple comparisons (999 permutations) were conducted, and the results were corrected using the false discovery rate (FDR) method to assess the robustness of each OPLS-DA model.

### 2.10 Differential expression analysis and identification of candidate metabolites

Differentially expressed metabolites (DEMs) were identified using the limma package (v 3.58.1) (32) with the criteria of |log_2_ Fold Change (FC)| > 1.0, *P* < 0.05, and VIP >1. The analysis was performed under two ion modes for specific group comparisons: In positive ion mode, comparisons between the model and control groups yielded DEMs1, and between the DHYZ and model groups yielded DEMs2. In negative ion mode, the corresponding comparisons produced DEMs3 (model vs. control) and DEMs4 (DHYZ vs. model). Volcano plots for DEMs1–DEMs4 were generated using the ggplot2 package (v 3.5.1), labeling the top 10 up- and down-regulated metabolites based on |log_2_ FC|.

Venn diagrams were constructed using the VennDiagram package (v 1.7.3) to identify metabolites with reversed expression trends. For the positive ion mode data, the intersection between up-regulated metabolites in DEMs1 and down-regulated metabolites in DEMs2 was defined as intersection metabolites 1. Conversely, the intersection between down-regulated metabolites in DEMs1 and up-regulated metabolites in DEMs2 was defined as intersection metabolites 2. The union of these two sets constituted candidate metabolites 1. A parallel analysis was performed on the negative ion mode data (DEMs3 and DEMs4) to identify intersection metabolites 3 and 4, the union of which formed candidate metabolites 2. The overall candidate metabolite set was defined as the union of candidate metabolites 1 and 2. The abundance profiles of these final candidate metabolites across all samples were visualized in a heatmap using the pheatmap package (v 1.0.12). To explore the KEGG pathways associated with the candidate metabolites, an enrichment analysis was performed using the MetaboAnalyst platform, with significance set at *P* < 0.05. The results were visualized as a bubble chart using ggplot2.

### 2.11 Microbiome sequencing and data pre-processing

Amplification targeted conserved 16S rDNA/ITS2 gene regions employing total genomic DNA as template material. Target sequences underwent 35-cycle amplification via one-step PCR utilizing Phusion High-Fidelity DNA Polymerase. Subsequently, a secondary PCR step facilitated attachment of indexed sequencing adapters for library construction. Amplicon libraries experienced purification through AxyPrep PCR Cleanup Kit, underwent quantification via Quant-iT PicoGreen dsDNA Assay Kit on the Promega QuantiFluor platform, and were combined in equimolar proportions. Paired-end sequencing (2 × 250 bp) was ultimately executed on an Illumina platform following established protocols. Raw sequence outputs underwent processing through the DADA2 pipeline, encompassing quality filtration, denoising, and paired-end read consolidation, succeeded by chimeric sequence elimination via Vsearch to yield amplicon sequence variant (ASV) tables. ASV taxonomic classification was accomplished by sequence alignment to the SILVA database (https://www.arb-silva.de/) employing the Mothur algorithm. Sequencing depth sufficiency was evaluated through rarefaction curve generation for all three-group samples using ggplot2 package (v 3.5.1), examining the correlation between observed species diversity and sequencing intensity.

### 2.12 Alpha and beta diversity analyses and species composition profiling

Alpha diversity, quantifying species richness, diversity, and evenness within localized environments, was determined by computing ACE, Chao1, Shannon, and invSimpson metrics via the vegan package (v 2.6.4). Inter-group statistical variations in these metrics across DHYZ, model, and control cohorts were assessed through Wilcoxon rank-sum testing, applying *P* < 0.05 significance criteria. Violin plots generated via ggplot2 (v 3.5.1) provided result visualization. Beta diversity, reflecting inter-community compositional variation, was examined using Bray-Curtis and Jaccard distance measures. Principal coordinates analysis (PCoA) facilitated dissimilarity visualization, while permutational multivariate analysis of variance (PERMANOVA) tested statistical significance of group clustering. Microbial community compositional profiling across cohorts involved calculating relative abundance of microbial taxa per sample. The ggplot2 package generated community composition bar charts displaying the top 10 most prevalent taxa at phylum and genus hierarchical levels. Additionally, genus-level taxonomic entities were compared among groups to discern group-specific or shared taxa, visualized through the Venn Diagram package (v 1.7.3).

### 2.13 Functional and linear discriminant analysis effect size (LEfSe) analyses

Microbial community functional variations among the three cohorts were forecasted based on KEGG pathway annotations using PICRUSt2 (https://github.com/picrust/picrust2). Inter-group disparities in predicted KEGG pathway relative abundances were evaluated via Kruskal-Wallis testing (*P* < 0.05). For identifying microbial taxa exhibiting significant differential prevalence across groups, Linear Discriminant Analysis Effect Size (LEfSe) methodology was implemented via the microeco package (v 1.8.0). This assessment employed significance criteria of *P* < 0.05 coupled with linear discriminant analysis (LDA) scores exceeding 2. Genera satisfying these statistical thresholds were designated as candidate microbes. LEfSe analytical outcomes were visualized through circular clustering diagrams produced with the circlize package (v 0.4.16) and bar charts created using ggplot2 (v 3.5.1).

### 2.14 Omics integration analysis

Spearman correlation analysis via the psych package (v 2.4.3) investigated associations between candidate metabolites and candidate microbes. Correlations exhibiting absolute coefficients (|ρ|) exceeding 0.30 with *P* < 0.05 were deemed statistically significant and displayed in correlation heatmaps generated through the ComplexHeatmap package (v 2.21.1).

To emphasize the most robust associations, more stringent criteria (|ρ| > 0.60, *P* < 0.05) were implemented to delineate subsets of key metabolites and key microbes. Sankey diagrams were subsequently constructed to depict interaction networks among these pivotal elements utilizing the ggsankey package (v 3.7.1).

Lastly, to discern biological pathways co-enriched by both key target genes (from antecedent analysis) and key metabolites, joint pathway examination was executed via the Joint-pathway module of the MetaboAnalyst platform (*P* < 0.05). Outcomes were rendered using the ggplot2 package (v 3.5.1).

### 2.15 Statistical analysis

R software (v 4.2.2) executed all statistical assessments. Inter-group comparisons employed either Wilcoxon rank-sum testing (two-group scenarios) or Kruskal-Wallis testing (multi-group scenarios). Statistical significance was established at P-values below 0.05.

## 3 Results

### 3.1 Results of retinal histopathological staining

**Figure 1:**
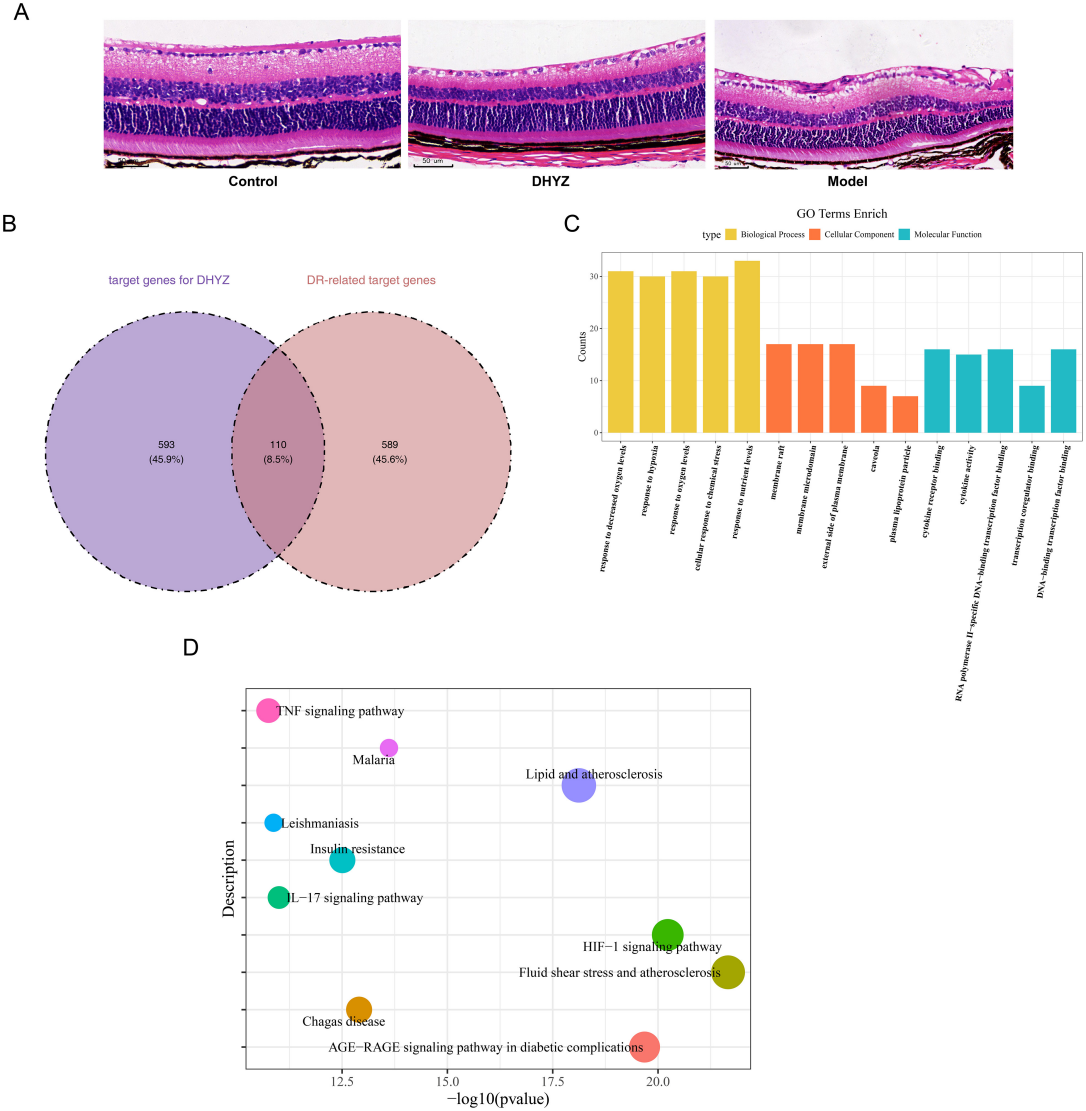
Histopathological staining confirms the therapeutic efficacy of DHYZ on DR, and network pharmacology reveals the multi-target mechanisms of DHYZ against DR. **(A)** Representative hematoxylin and eosin (HE) staining images of retinal tissue (Magnification × 400; Scale bar = 50 μm). **(B)** Venn diagram showing 110 candidate genes from the intersection of 703 DHYZ-related targets and 699 DR-related targets. **(C)** Gene Ontology (GO) enrichment analysis. **(D)** Kyoto Encyclopedia of Genes and Genomes (KEGG) pathway enrichment analysis.

In the control group, the retinal tissue exhibited well-defined laminar architecture, compact cellular arrangement, and showed no signs of edema or vascular abnormalities. In contrast, the model group displayed marked retinal edema, a reduction in ganglion cells, increased spacing with sparse cellularity in the outer nuclear layer, disorganized cell arrangement, and the presence of neovascularization (Figure 1A).

### 3.2 Recognition of 703 target genes for DHYZ

The active compounds of the 12 herbs in DHYZ were identified through the TCMSP, with the following numbers of compounds: 76 for Shudihuang, 174 for Baji-tian, 226 for Shanzhuyu, 75 for Roucongrong, 65 for Fuzi, 169 for Wuweizi, 100 for Rougui, 34 for Fuling, 105 for Shichangpu, 164 for Bohe, 265 for Shengjiang, and 133 for Dazao. A total of 556 target genes 1 were then retrieved from the Uniprot database.

In addition, the HERB database provided data for 3 additional herbs: 58 active compounds and 158 target genes for Shi Hu, 55 active compounds and 3 target genes for Mai Dong, and 101 active compounds and 108 target genes for Yuan Zhi yielding a total of 235 target genes 2. The intersection of the 556 target genes 1 and the 235 target genes 2 resulted in 703 target genes for DHYZ.

### 3.3 Identification and enrichment analysis of 110 candidate genes

Following explorations in the OMIM and TTD databases, 234 and 30 target genes associated with DR were identified, respectively. The 234 target genes from the OMIM database, 30 target genes from the TTD database, and 500 genes from the Genecards database were merged and deduplicated, yielding 699 DR-related target genes. Subsequently, the intersection of 703 target genes for DHYZ and 699 DR-related target genes resulted in 110 candidate genes (Figure 1B).

Further analysis of these 110 candidate genes using GO analysis identified 2,584 terms (*P* < 0.05), including 2,331 BP terms, 77 CC terms, and 176 MF terms. At the GO level, these candidate genes were primarily associated with functions such as “response to decreased oxygen levels” (BP), “membrane microdomain” (CC), and “cytokine activity” (MF) (*P* < 0.05) (Figure 1C). Moreover, 160 KEGG pathways were identified, including the “IL-17 signaling pathway”, “HIF-1 signaling pathway”, and “AGE-RAGE signaling pathway in diabetic complications” (*P* < 0.05) (Figure 1D). These analyses provided a strong foundation for understanding the functional importance of candidate genes in the progression of DR.

### 3.4 Selection of 5 key target genes

**Figure 2:**
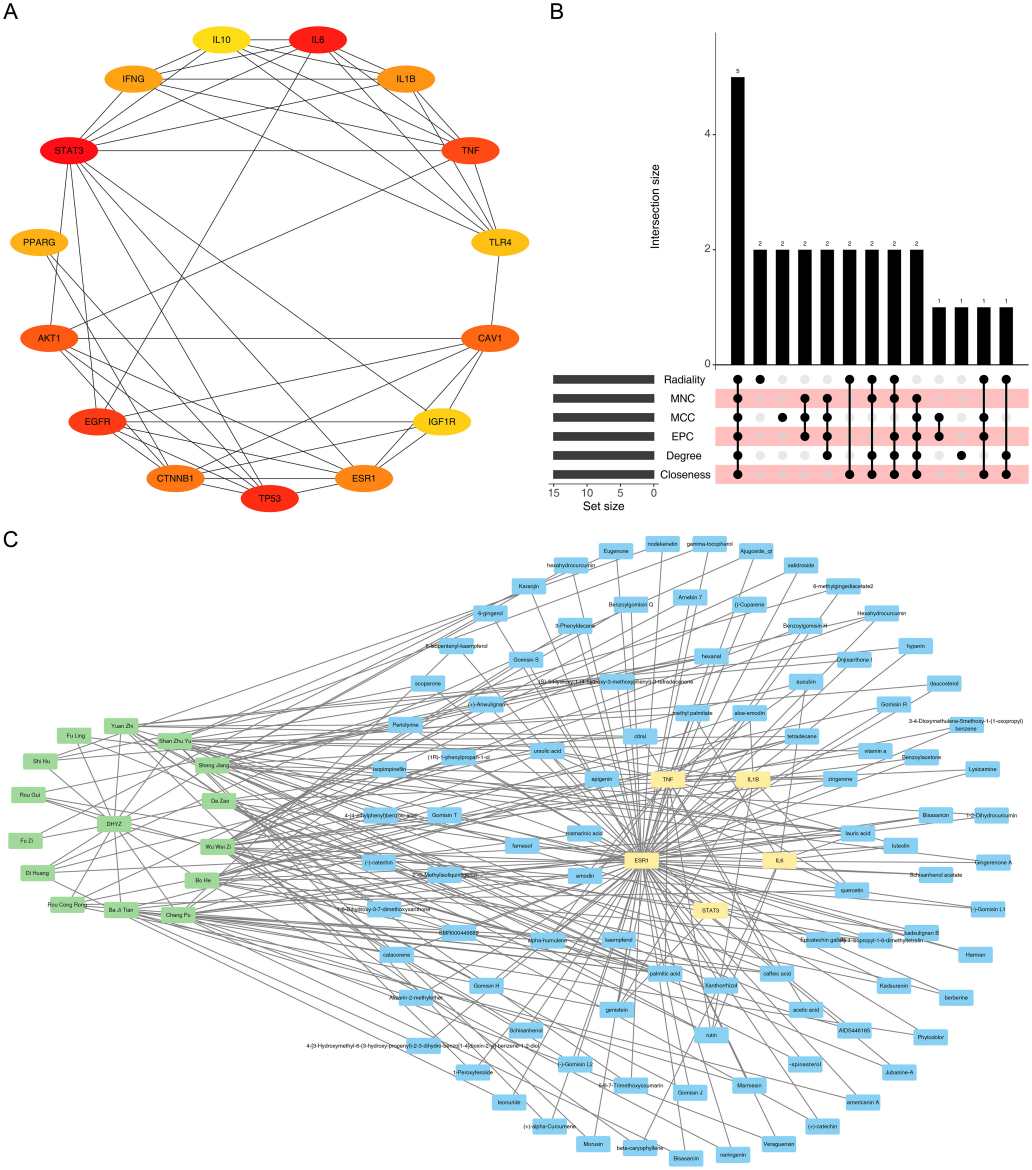
Identification of key targets and construction of the compound-target-herb network. **(A)** Protein-protein interaction (PPI) network of the top 15 candidate genes. Each node represents a protein, and the size of the node usually reflects its degree value, while the thickness of the lines typically indicates the confidence level or strength of the interaction. The node labels directly indicate the official gene symbols of the proteins (such as IL10, ESR1, etc). **(B)** Identification of 5 key target genes based on the overlap from six topological analysis methods. This figure consists of two parts. The left part shows the number of genes in each set, while the right part indicates the number of genes that are common to the combinations of different algorithms. **(C)** Integrated network diagram incorporating key targets, their corresponding active compounds, and related herbal constituents. The green nodes represent traditional Chinese medicines, the blue nodes represent active ingredients, and the yellow nodes represent candidate target genes.

The PPI network of the top 15 candidate genes revealed that STAT3, EGFR, and TP53 were hub nodes with extensive connections (Figure 2A). Five key target genes (STAT3, IL6, TNF, ESR1, and IL1B) were subsequently identified as the consensus core set by intersecting the top-ranked genes from six distinct topological algorithms (Figure 2B). To visualize the relationships between these key targets, their corresponding active compounds, and the source herbs, a comprehensive “compound-target-herb” network was constructed (Figure 2C).

### 3.5 Molecular docking analysis of ESR1 with bioactive compounds

**Figure 3:**
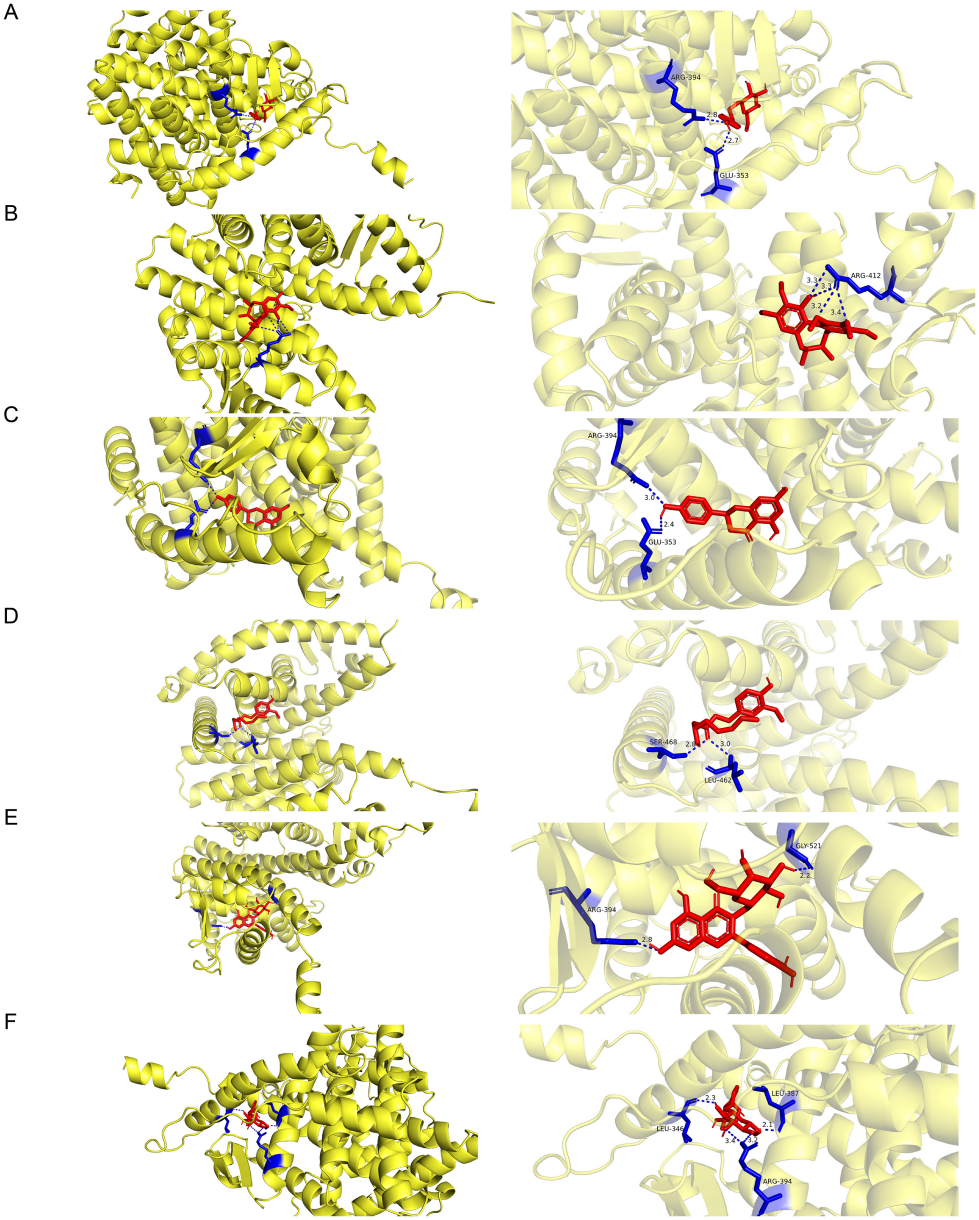
Molecular docking analysis reveals interactions between ESR1 and active compounds. **(A–F)** Binding modes of ESR1 with salidroside **(A)**, schisanhenol **(B)**, naringenin **(C)**, 6-gingerol **(D)**, hyperin **(E)**, and leonuride **(F)**, respectively. (The protein is depicted as a yellow cartoon structure, while the compound is represented by red stick models).

Molecular docking was performed to characterize the binding interactions between the protein ESR1 and six corresponding active compounds (salidroside, schisanhenol, naringenin, 6-gingerol, hyperin, and leonuride). All six compounds exhibited strong binding affinities to ESR1, with naringenin showing the highest affinity (Table 2, Figures 3A–F). The detailed binding parameters are as follows: Naringenin displayed the strongest binding energy (-8.9 kcal/mol), forming hydrogen bonds with residues ARG-394 and GLU-353 (Figure 3C). Salidroside bound at −8.1 kcal/mol, interacting via hydrogen bonds with ARG-394 and GLU-353 (Figure 3A). Hyperin and leonuride showed binding energies of −7.9 kcal/mol and −7.8 kcal/mol, interacting with residues ARG-394/GLY-521 and ARG-394/LEU-346/LEU-387, respectively (Figures 3E, F). Schisanhenol and 6-gingerol had binding energies of −6.7 kcal/mol and −5.6 kcal/mol, forming hydrogen bonds with ARG-412 and LEU-462/SER-468, respectively (Figures 3B, D). These results confirm robust binding between ESR1 and the bioactive compounds, supporting their potential functional relevance.

**Table 2:** Binding energies of ESR1 and 6 active compounds.

Gene	Active compounds	Binding energy
ESR1	salidroside	−8.1 kcal/mol
ESR1	Schisanhenol	−6.7 kcal/mol
ESR1	naringenin	−8.9 kcal/mol
ESR1	6-gingerol	−5.6 kcal/mol
ESR1	hyperin	−7.9 kcal/mol
ESR1	leonuride	−7.8 kcal/mol

### 3.6 Quality assessment of metabolomic profiles

**Figure 4:**
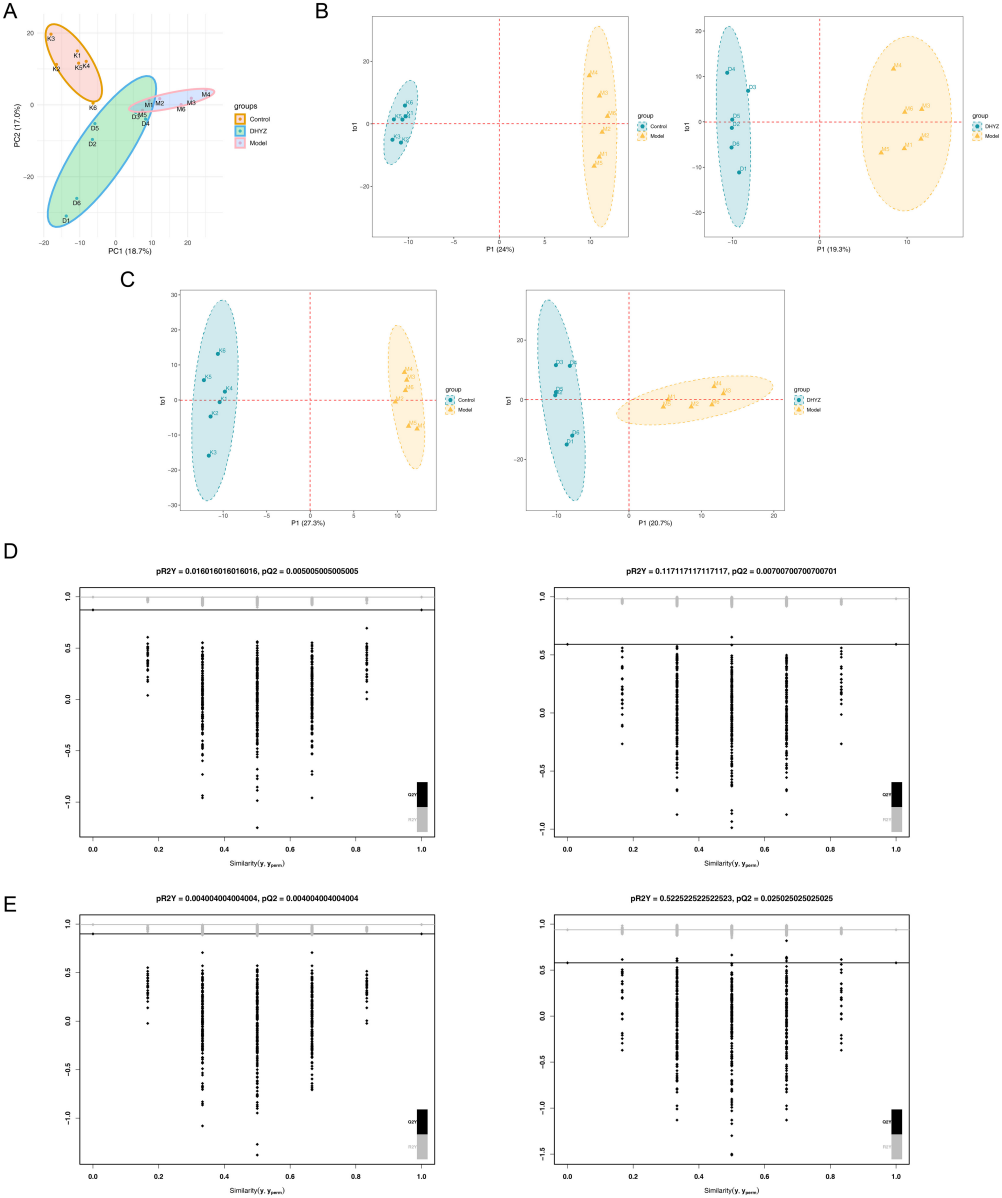
Metabolomic data quality assessment demonstrates model stability and reliability. **(A)** PCA plot showing clear separation among control, model, and DHYZ groups. **(B, C)** Orthogonal projections to latent structures-discriminant analysis (OPLS-DA) score plots. **(D, E)** Permutation test results validating the OPLS-DA models.

PCA showed clear separations among the control, model, and DHYZ groups, indicating distinct metabolic profiles (Figure 4A). OPLS-DA further demonstrated clear metabolic separations between the model and control groups, and between the DHYZ and model groups, in both positive and negative ion modes (Figures 4B, C). The robustness of the OPLS-DA models was validated by permutation tests (999 iterations), which confirmed the models were statistically significant and not overfitted (Figures 4D, E). The distinct group separations and validated models demonstrate the high quality and reliability of the metabolomic data, providing a solid foundation for subsequent analyses.

### 3.7 Identification and functional exploration of candidate metabolites

**Figure 5:**
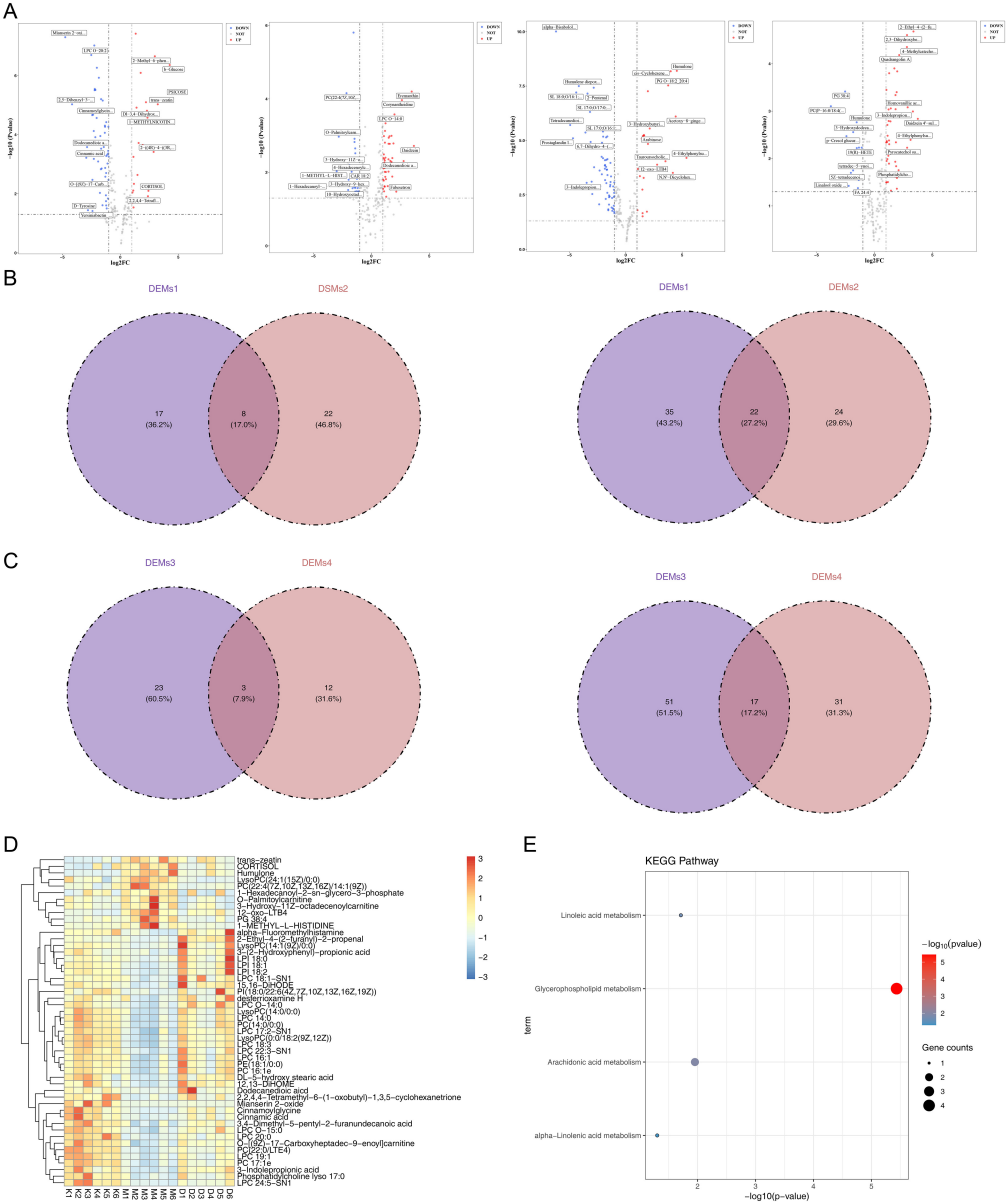
Metabolite identification and functional analysis. **(A)** Volcano plot illustrating up- and down-regulated differential metabolites. Each point in the figure represents a metabolite. The red points represent up-regulated metabolites, the blue points represent down-regulated metabolites, and the gray points represent metabolites with no significant difference. **(B)** The intersection situation of up-regulation and down-regulation for MC1 and DM1. MCU and MCD, respectively represent the up-regulated genes and down-regulated genes of MC1; DMU and DMD, respectively represent the up-regulated and down-regulated genes of DM1. **(C)** The intersection situation of up-regulation and down-regulation of NMC and NDM. NMCU and NMCD, respectively represent the up-regulated genes and down-regulated genes of NMC; NDMU and NDMD, respectively represent the up-regulated genes and down-regulated genes of NDM. **(D)** Expression heat map of key metabolites. The horizontal axis represents the samples, and the vertical axis represents the key metabolites. The darker the color, the higher the expression level of the metabolite in that sample. **(E)** KEGG pathway enrichment analysis bubble chart for key metabolites. The vertical axis represents the description of each pathway, and the larger the circle, the more key metabolites are enriched in that pathway.

Differential expression analysis (thresholds: |log_2_FC| > 1.0, *P* < 0.05, VIP > 1) identified 82 DEMs1 [25 up-, 57 down-regulated, Differential metabolite 1 (MC1)], 76 DEMs2 [46 up-, 30 down-regulated, Differential metabolite 2 (DM1)], 94 DEMs3 [26 up-, 68 down-regulated, Differential metabolite 3 (NMC)], and 63 DEMs4 [48 up-, 15 down-regulated, Differential metabolite 4 (NDM)] in the respective group comparisons (Figure 5A). Venn analysis was used to identify metabolites with reversed expression trends. In the positive ion mode, the intersection of DEMs1 and DEMs2 yielded 8 intersection metabolites 1 and 22 intersection metabolites 2, which were combined into 30 candidate metabolites 1 (Figure 5B). Similarly, analysis of DEMs3 and DEMs4 in the negative ion mode identified 3 intersection metabolites 3 and 17 intersection metabolites 4, together forming 20 candidate metabolites 2 (Figure 5C). The union of these two sets resulted in a final list of 50 candidate metabolites, whose expression patterns across the three groups are displayed in a heatmap (Figure 5D).

Enrichment analysis of the 50 candidate metabolites revealed four significant KEGG pathways (*P* < 0.05): arachidonic acid metabolism, alpha-Linolenic acid metabolism, glycerophospholipid metabolism, and linoleic acid metabolism (Figure 5E). These pathways are implicated in critical metabolic and signaling processes, suggesting their potential involvement in the condition under study.

### 3.8 Microbiome sequencing depth assessment

**Figure 6:**
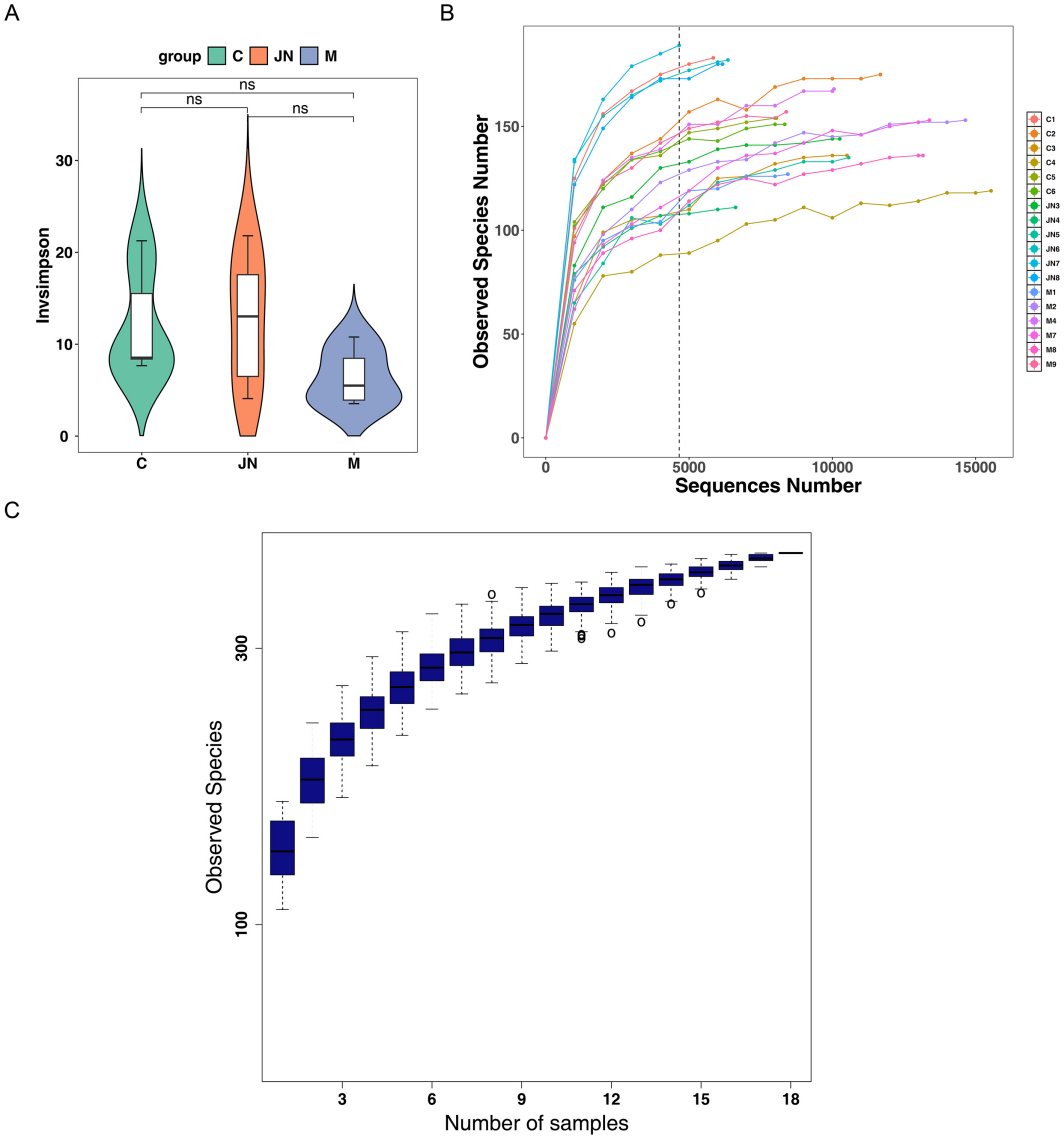
Microbiome analysis results. **(A)** InvSimpson diversity index. **(B)** Species rarefaction curve, reflecting the relationship between sequencing depth and the completeness of species detection. **(C)** Box plot illustrating the impact of sample size on the completeness of species diversity detection.

To evaluate the adequacy of microbiome sequencing depth, rarefaction curves were analyzed. The asymptotic approach of the curves indicated that sufficient depth was achieved, as further sequencing would unlikely yield substantial new amplicon sequence variants (ASVs) (Figure 6B). This was supported by the species accumulation boxplot, which showed that observed species richness increased with sampling effort and began to plateau (Figure 6C). Together, these results confirm that sequencing depth was adequate to capture the microbial diversity and reveal clear differences among the three groups.

### 3.9 Analysis of alpha and beta diversity

**Figure 7:**
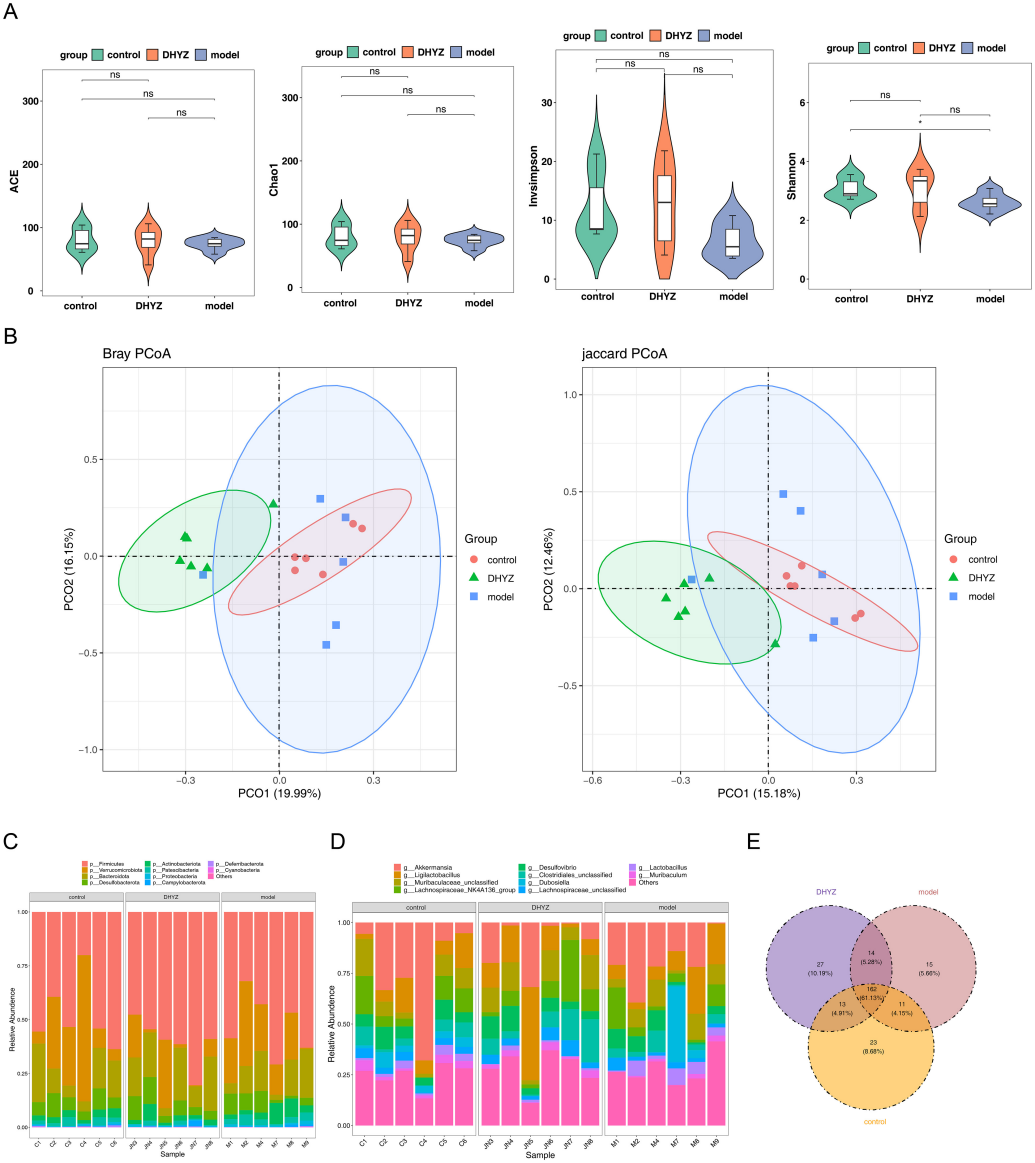
Gut microbiota composition and diversity analysis. **(A)** Assessment of α-diversity indices, including ACE, Chao1, Shannon, and inverse Simpson. **(B)** β-diversity analysis based on principal coordinate analysis (PCoA). **(C, D)** Microbial composition at the phylum and genus levels, respectively, across the three groups. **(E)** Venn diagram showing the distribution of genera among the three groups.

Alpha diversity metrics including ACE, Chao1, Shannon, and invSimpson indices were employed for assessment. The Shannon index exhibited statistically significant variation between model and control cohorts (*P* < 0.05) (Figure 6A), whereas remaining indices showed no statistical significance across all group comparisons (*P* > 0.05) (Figure 7A). Beta diversity examination via PCoA plots demonstrated an absence of distinct microbial community structure clustering among the three experimental groups (Figure 7B).

Microbial composition analysis at the phylum and genus levels showed that Firmicutes was the dominant phylum, and Akkermansia was the most abundant genus across all groups (Figures 7C, D). Venn analysis of genus-level taxa identified 162 microbes shared among the three groups, while the control, model, and DHYZ groups contained 23, 15, and 27 unique microbes, respectively (Figure 7E). These results indicate distinct patterns in microbial diversity and composition among the groups, providing insight into potential microbiome-related mechanisms.

### 3.10 Distinct microbial signatures among the 3 groups

**Figure 8:**
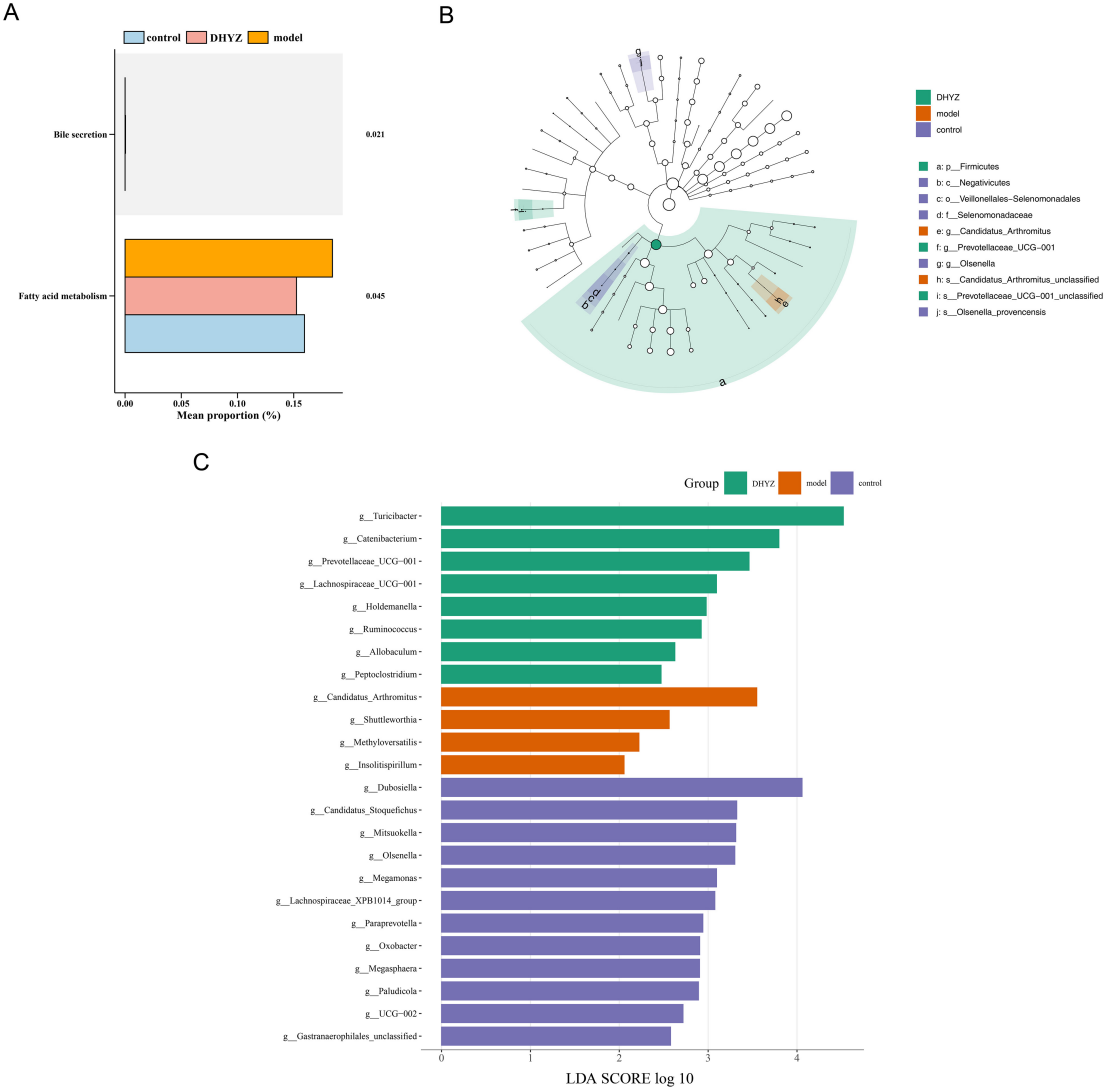
Functional and phylogenetic characterization of gut microbiota. **(A)** Differential abundance of predicted functional pathways related to bile secretion and fatty acid metabolism among groups. **(B)** Cladogram illustrating phylogenetic distribution of microbial taxa across groups. **(C)** Linear discriminant analysis (LDA) effect size (LEfSe) identifying differentially abundant microbial taxa.

Further analysis of the microbes among the 3 groups using PICRUSt2 analysis identified 2 KEGG pathways, including “fatty acid metabolism” and “bile secretion” (*P* < 0.05) (Figure 8A). These findings suggested that specific metabolic pathways might play a crucial role in distinguishing the microbial profiles. Moreover, LEfSe analysis revealed 24 candidate microbes at the genus level among the 3 groups (*P* < 0.05, LDA score >2) (Figures 8B, C). These results underscore the potential functional and taxonomic differences that might contribute to the microbial signature, providing insights into the underlying mechanisms.

### 3.11 Identification of distinct microbial signatures

Functional prediction via PICRUSt2 revealed two significantly enriched KEGG pathways (*P* < 0.05): “fatty acid metabolism “and” bile secretion” (Figure 8A). In addition, LEfSe analysis identified 24 differentially abundant genera (LDA score >2, *P* < 0.05) as candidate microbes distinguishing the three groups (Figures 8B, C). These results indicate distinct functional and taxonomic microbial signatures among the groups, suggesting potential mechanistic differences.

### 3.12 Strong correlations between candidate metabolites and candidate microbes

**Figure 9:**
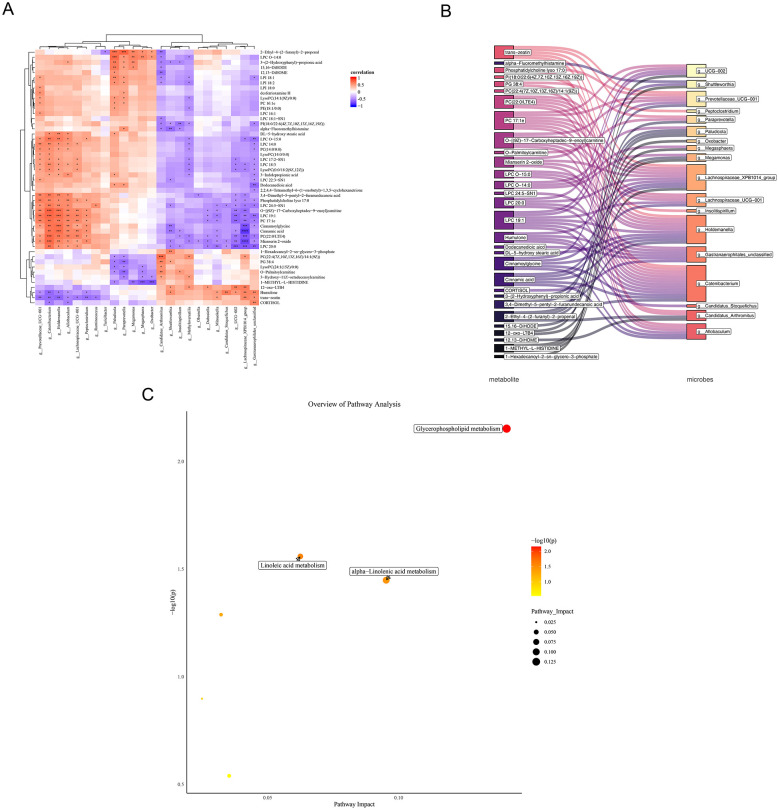
Integrated microbe-metabolite correlation and pathway analysis. **(A)** Heatmap displaying Spearman correlations between key microbes and metabolites. **(B)** Chord diagram visualizing metabolite-microbe associations. **(C)** Bubble plot of KEGG pathway enrichment analysis.

Employing the previously discerned 50 candidate metabolites and 24 candidate microbes, an investigation into the symbiotic interplay between metabolites and microbes was conducted. The results revealed that cinnamic acid showed the strongest negative correlation with Lachnospiraceae_XPB1014_group (cor = −0.86, *P* < 0.0001), while O-[(9Z)-17-Carboxyheptadec-9-enoyl] carnitine exhibited the strongest positive correlation with Catenibacterium (cor = 0.77, *P* = 0.0002) (Figure 9A). Furthermore, 30 key metabolites and 18 key microbes were selected with |cor| > 0.60 and *P* < 0.05 as the selection criteria, and a regulatory network was constructed to elucidate the correlations between these key metabolites and key microbes (Figure 9B). Additionally, the metabolic pathway analysis of the 5 key target genes and 30 key metabolites revealed 3 commonly enriched pathways, including “glycerophospholipid metabolism”, “linoleic acid metabolism”, and “alpha-linolenic acid metabolism” (Figure 9C). These findings unveiled the complex interplay between metabolites and microbes, while also highlighting the involvement of key target genes and key metabolites in metabolic pathways, providing new insights for further investigation into metabolic regulation.

### 3.13 Correlations between candidate metabolites and candidate microbes

To investigate the interplay between metabolites and microbes, we performed a Spearman correlation analysis using the 50 candidate metabolites and 24 candidate microbes. Cinnamic acid showed the strongest negative correlation with *Lachnospiraceae_XPB1014_group* (ρ = −0.86, *P* < 0.0001), while O-[(9Z)-17-Carboxyheptadec-9-enoyl]carnitine exhibited the strongest positive correlation with *Catenibacterium* (ρ = 0.77, *P* = 0.0002) (Figure 9A). Applying stricter criteria (|ρ| > 0.60, *P* < 0.05), we identified 30 key metabolites and 18 key microbes, and constructed a correlation network to visualize their interactions (Figure 9B). Furthermore, joint pathway analysis of the 5 key target genes and these 30 key metabolites revealed three commonly enriched pathways: glycerophospholipid metabolism, linoleic acid metabolism, and alpha-linolenic acid metabolism (Figure 9C). These results elucidate the complex metabolite-microbe interactions and implicate key targets in specific metabolic pathways, providing new insights into underlying regulatory mechanisms.

## 4 Discussion

As a predominant microvascular complication of diabetes, DR substantially increases the risk of blindness in the working-age cohort (2). The pathogenic mechanisms of DR are multifaceted, involving synergistic yet distinct signaling cascades that are imperfectly deciphered (33). Given the limited efficacy of single-target therapeutic approaches in addressing such complexity, emerging evidence suggests that TCM holds considerable promise in DR management through its multi-target, multi-pathway regulatory effects (34). Our preliminary data demonstrated that DHYZ attenuates retinal impairment in DR, potentially through the activation of PI3K/Akt and MAPK signaling pathways (17). In the present study, a combinatorial approach incorporating network pharmacology, metabolomics, and gut microbiota analysis was utilized to independently pinpoint pivotal targets, candidate metabolites, and candidate microbes. Subsequent molecular docking and functional enrichment analyses were conducted to interrogate the biological interplay among these elements. Through integrative multi-omics analysis, 30 critical metabolites and 18 key microbes were discerned. By synthesizing multi-tiered biological intelligence, this strategy facilitates a systems-level examination of gene functions and regulatory networks, thereby elucidating the intricate molecular crosstalk and signaling pathways implicated in DR (35, 36). Network analysis of the “DHYZ—Active Ingredients—Targets” network identified six primary active components in DHYZ: salidroside, schisanhenol, naringenin, 6-gingerol, hyperin, and leonuride.

Among the six identified active components, salidroside (SAL), a phenolic glycoside, has attracted considerable attention for its antioxidant and anti-inflammatory properties, which are particularly relevant to diabetic complications. Beyond its reported effects in inhibiting tumor growth, delaying aging, and alleviating oxidative stress (37–41), SAL shows specific therapeutic potential in diabetes-related pathologies. Most importantly, SAL has been demonstrated to ameliorate DR by reducing Müller cell inflammation via the PI3K/Akt/GSK-3β/NF-κB pathway (42). Additionally, SAL mitigates STZ-induced neuropathic pain in diabetic rats by suppressing spinal cord neuroinflammation (43), suggesting its broad protective effects against diabetes complications.

Schisanhenol, a natural compound derived from Schisandra chinensis, has been shown to modulate glucose metabolism. Treatment with Schisandra extracts containing schisanhenol reduced hepatic and muscular glycogen content by up to one-third in mice (44), suggesting potential effects on glucose homeostasis that may be relevant to diabetic metabolic dysregulation. Naringenin, a natural flavonoid with diverse biological activities including antibacterial, anti-inflammatory, antioxidant, immunomodulatory, and antitumor effects (45, 46), has shown efficacy against cardiovascular disease (47), atherosclerosis (48), and diabetes (49). Particularly relevant to DR pathogenesis, naringenin alleviates high glucose-induced vascular endothelial cell injury (50), a key pathological event in retinal microvascular dysfunction.

6-Gingerol, a bioactive component of ginger, significantly reduces hyperglycemia, retinal vascular diameter, and basement membrane thickness (51). Ginger extract modulates NF-κB expression and markedly lowers TNF-α and VEGF levels in retinal tissues (52). Hyperin, a major active constituent of A. manihot, ameliorates retinal damage in DR rats by modulating the TGF-β1/miR-200b/VEGF signaling pathway (53). Lastly, leonuride, an alkaloid from Leonurus japonicus, exhibits antioxidant and anti-inflammatory properties and confers protection against diabetic nephropathy by activating the SREBP pathway (54). Collectively, these six active components of DHYZ exert their therapeutic effects through multiple mechanisms, including antioxidant defense, anti-inflammatory action, metabolic regulation, and modulation of key signaling pathways. This multi-component, multi-target characteristic is consistent with the holistic therapeutic principle of traditional Chinese medicine and may explain the synergistic efficacy of DHYZ in treating DR.

To identify the molecular targets underlying DHYZ's therapeutic effects, we integrated network pharmacology analysis with protein-protein interaction networks, which led to the identification of five key targets: STAT3, IL6, TNF, ESR1, and IL1B STAT3 is a transcription factor involved in cell proliferation, differentiation, apoptosis, and immune responses, and serves as a critical mediator of cytokine signaling. Accumulating evidence supports its central role in the pathogenesis and treatment of DR. Notably, traditional Chinese medicine has been reported to alleviate oxidative stress-induced retinal injury by suppressing STAT3 expression. IL-6 (55–58), IL1B (59–61), and TNF (61–64) are cytokines pivotal in inflammatory processes. They participate in immune and inflammatory responses, promote acute inflammation via NF-κB pathway activation, and contribute to the pathogenesis of several chronic diseases. Clinical studies have indicated that ocular levels of IL-1β, TNF-α, IL-8, and IL-6 are closely associated with DR progression (65–68). The ESR1 gene encodes estrogen receptor alpha (ERα), which regulates gene expression upon estrogen binding and participates in key physiological processes such as reproduction, bone metabolism, and cardiovascular function (69, 70). Intriguingly, FAM209B and PTGES have been proposed as immune-related biomarkers in DR, and ESR1 may transcriptionally regulate both genes, suggesting its potential involvement in DR pathogenesis.

In this study, we utilized metabolomics to identify differentially expressed metabolites. Pathway enrichment analysis revealed that the differentially expressed metabolites converged on four interconnected lipid metabolism pathways (*P* < 0.05): arachidonic acid metabolism, α-linolenic acid metabolism, linoleic acid metabolism, and glycerophospholipid metabolism. These pathways collectively drive DR pathogenesis through inflammatory mediator production, oxidative stress amplification, and cellular membrane dysfunction. First, dysregulated arachidonic acid metabolism may lead to excessive production of pro-inflammatory mediators (e.g., prostaglandins and leukotrienes), promoting early microvascular inflammation and blood–retinal barrier disruption. Concurrently, alterations in α-linolenic acid metabolism (71, 72) and linoleic acid metabolism (73) reflect an imbalance in polyunsaturated fatty acid homeostasis, potentially exacerbating DR through oxidative stress, neurovascular injury, and impaired synthesis of pro-resolving mediators. Furthermore, aberrant glycerophospholipid metabolism (74) compromises cellular membrane architecture, consequently disrupting membrane-associated signaling platforms and promoting retinal cell apoptosis. Collectively, these lipid metabolism pathways form an interconnected network that drives DR pathogenesis through coordinated inflammatory responses, oxidative damage, and cellular membrane dysfunction.

Accumulating evidence has highlighted the critical role of gut microbiota in diabetes and its complications, including DR, through the gut-retina axis (75). To investigate these microbial alterations, we utilized STZ-induced diabetic mice, which exhibited significant gut microbiota dysbiosis at multiple taxonomic levels. We comprehensively assessed microbial community characteristics using multiple alpha diversity indices (ACE, Chao1, Shannon, and Simpson) to evaluate species richness and diversity. Together, these metrics indicated disrupted gut microbiota in diabetic mice, characterized by altered richness, diversity, and composition. Although DHYZ intervention did not fully reverse these changes, it promoted a partial shift toward a more normalized microbial structure.

Studies have established a correlation between obesity in diabetic patients and gut microbiome composition, with the Firmicutes-to-Bacteroidetes (F/B) ratio often proposed as a potential indicator of metabolic dysregulation. In our study, the F/B ratio was highest in the model (M) group, consistent with observations in T2DM patients (76), where an elevated ratio is linked to enhanced energy harvest and metabolic disease progression. However, some reports associate disease progression with increased Bacteroidetes abundance rather than the F/B ratio (77), suggesting that factors such as diet, environment, physical activity, and socioeconomic status may contribute to these discrepancies. Notably, DHYZ treatment significantly reduced the F/B ratio in the JN group, indicating that modulation of gut microbiota composition may be one mechanism by which it alleviates DR symptoms.

At the class level, an increase in Bacilli was observed in the model group. Together with the elevated F/B ratio, the increased abundance of Bacilli—a class enriched in polysaccharide-degrading taxa capable of extracting more energy from dietary components—suggests enhanced energy harvest efficiency and subsequent fat accumulation. This finding is consistent with the obese phenotype observed in db/db mice and supported by clinical data (78, 79). Furthermore, a reduction in butyrate-producing Clostridia was noted. Butyrate is crucial for maintaining gut barrier integrity, suppressing inflammation, and improving insulin sensitivity (80–82). A decline in Clostridia may lead to decreased butyrate production, thereby impairing gut barrier function and exacerbating systemic inflammation and insulin resistance, which aligns with our experimental findings.

Functional analysis revealed significant disruptions in microbial KEGG pathways related to “fatty acid metabolism” and “bile secretion” in the model group. DHYZ intervention substantially ameliorated these pathway disruptions, restoring them toward normal levels. The mechanism may involve the suppression of fatty acid metabolism in diabetes, reducing short-chain fatty acid (SCFA) production and compromising intestinal barrier function and insulin sensitivity. Concurrently, dysregulated bile secretion, coupled with altered microbial bile acid transformation, may lead to abnormal accumulation of secondary bile acids. This dysregulation can result in aberrant FXR pathway activation, thereby exacerbating glucose and lipid metabolic disorders. DHYZ appears to counteract these effects by remodeling gut microbiota function—promoting SCFA production and inhibiting bile acid transformation—thereby ameliorating host metabolic and inflammatory status. These findings illustrate how DHYZ may intervene in DR through the microbiota-metabolism axis, restoring intestinal microecological homeostasis and providing a theoretical basis for the multi-target regulatory effects of traditional Chinese medicine.

A strong negative correlation was observed between cinnamic acid and the gut bacterium Lachnospiraceae_XPB1014_group. Cinnamic acid, an aromatic compound with demonstrated antioxidant, anti-inflammatory, antimicrobial, and metabolic regulatory properties (83, 84), protects against oxidative damage by scavenging intracellular free radicals and suppressing pro-inflammatory cytokines. It has been shown to ameliorate endothelial dysfunction-related disorders, including obesity and diabetes (85). Lachnospiraceae is a major family of anaerobic gut bacteria, and taxa within the XPB1014_group are considered beneficial, contributing to gut health through the production of SCFAs like butyrate. SCFAs are crucial for maintaining intestinal barrier integrity, reducing inflammation, and modulating immune responses (86, 87). Following the direct and significant anti-inflammatory effect exerted by cinnamic acid, the host's requirement for inflammation resolution mediated by butyrate production by this bacterial community is diminished, which indirectly results in the downregulation of its abundance, thus generating a strong negative correlation between the two.

Conversely, a strong positive correlation was identified between O-[(9Z)-17-Carboxyheptadec-9-enoyl]carnitine and Catenibacterium. This metabolite is an acylcarnitine ester, which plays a key role in fatty acid metabolism by transporting fatty acids into mitochondria for β-oxidation and energy production. Acylcarnitines are emerging as potential biomarkers in ocular diseases (88).

The genus Catenibacterium, an obligate anaerobic bacterium within the gut microbiota (89), primarily ferments dietary fibers and complex polysaccharides to produce SCFAs such as butyrate. These SCFAs contribute to intestinal barrier integrity, inflammatory modulation, promotion of beneficial microbial communities, and overall metabolic homeostasis (90).

Integrated metabolic pathway analysis of the five key target genes and thirty critical metabolites revealed significant co-enrichment in three key pathways: glycerophospholipid metabolism, linoleic acid metabolism, and α-linolenic acid metabolism (91, 92). These pathways are essential for maintaining cellular structure, signal transduction, immune regulation, and cardiovascular and neurological function. Specifically, glycerophospholipid metabolism supports membrane integrity and signaling processes; linoleic acid metabolism regulates inflammation and lipid homeostasis; and α-linolenic acid metabolism contributes to cardiovascular and cognitive health via its anti-inflammatory derivatives. These findings not only outline a complex metabolite–microbe interaction network but also clarify the synergistic involvement of key targets and metabolites in metabolic pathways, offering new theoretical foundations for metabolic regulation research.

The five key target genes (STAT3, IL6, TNF, ESR1, IL1B), 30 key metabolites, and 18 key microorganisms identified in this study do not function in isolation, but may constitute a multi-level synergistic regulatory network via the gut-retina axis (93, 94). At the microbial level, active ingredients of DHYZ (such as naringenin) remodel the structure of the gut microbiota (95). Changes in these microbiota directly alter their metabolic output, generating key metabolites that reach the retinal microenvironment through the circulatory system and act as “messengers” linking the gut and the retina (96, 97). At the target gene level, these metabolites (e.g., short-chain fatty acids) modulate the expression of immune-related genes via G-protein-coupled receptors (98), further regulating the activity of inflammatory pathways such as STAT3 and NF-κB in retinal cells and influencing the expression of target genes including ESR1, IL6, TNF, and IL1B. This ultimately determines the integrity of the blood-retinal barrier and the functional status of the neurovascular unit.

This network exhibits synergistic features of multi-node crosstalk and multi-feedback regulation: microbial metabolites directly regulate target gene expression, while alterations in target genes (such as ESR1) can in turn feedback on gut permeability and microbiota composition through neuroendocrine pathways, forming a closed-loop regulatory system. Combined pathway analysis confirmed that lipid metabolic pathways, including glycerophospholipid metabolism and linoleic acid metabolism, serve as pivotal hubs connecting the three core components (74, 99). Their dysregulation is both a consequence of target gene modulation and a source of metabolite alterations (100). This systematic integrative analysis reveals the potential mechanism by which DHYZ coordinately maintains homeostasis of the gut-retina axis through a three-level microbe-metabolite-target gene network. It also provides a clear direction for subsequent functional validation experiments—such as fecal microbiota transplantation and gene knockout—focusing on the causal relationships among key microbiota, metabolites, and target genes.

In summary, this study innovatively employed an integrated multi-omics strategy combining network pharmacology, metabolomics, and gut microbiota analysis to systematically elucidate the potential mechanisms of DHYZ) in treating DR—an approach rarely applied in previous investigations of multi-target mechanisms of TCM formulas in the context of DR. Through this strategy, we identified, for the first time, that the core regulatory elements of DHYZ's therapeutic effects include five key target genes, 30 critical metabolites, and 18 key microbial taxa. These findings not only provide important evidence for understanding the “host–microbiota–metabolite” interactive network in DR pathogenesis but also open new avenues for subsequent research. Nevertheless, several limitations exist in the present study. First, in terms of experimental design, only a single dose of DHYZ was administered, without multiple dose groups to systematically evaluate its dose–effect relationship. This restricts our determination of the optimal therapeutic window and precludes the revelation of associations between dose-dependent efficacy and target regulation. Second, we mainly relied on hematoxylin and eosin (HE) staining to observe he basic histological morphology of the retina, without detecting DR-specific pathological phenotypes; thus, the histological evidence is insufficient to fully substantiate the therapeutic efficacy of DHYZ. Third, this study was primarily based on bioinformatic analysis, lacking molecular biological experiments to functionally validate key targets (e.g., ESR1) and core metabolites. Although molecular docking predicted the binding potential of active ingredients (e.g., naringenin, salidroside) to targets such as ESR1, virtual screening cannot substitute for functional verification *in vitro* or *in vivo*. In addition, the small sample size (*n* = 6) and exclusive use of the db/db mouse model make it difficult to fully recapitulate the complex pathological progression of human DR. The multi-omics integrative analysis was predominantly correlational, failing to clarify the causal regulatory network among the host, microbiota, and metabolites; meanwhile, microbial function prediction was limited by the completeness of current databases.

Future research should first validate the stability and reproducibility of key targets, metabolites, and microbiota using multicenter clinical cohorts or expanded animal model sample sizes. Multi-dose studies should be performed to integrate pharmacodynamic, metabolomic, and microbiomic data for a systematic assessment of the dose-response relationship of DHYZ in the treatment of DR. Combined with pharmacokinetics, the exposure-effect relationship of its active ingredients should be clarified to guide precise clinical medication. On this basis, strategies including intestinal-specific ESR1 knockout mice, monocolonization experiments of key microbiota, and targeted metabolomics can be adopted to further verify the mechanism of the gut-retina axis in DHYZ-mediated intervention against DR. Meanwhile, functional validation at the cellular level should be conducted for the predicted interactions between active ingredients and key targets (e.g., ESR1) via molecular docking: using gene silencing/overexpression combined with active ingredient intervention to detect their effects on downstream inflammatory pathways and cellular functions, thereby converting virtual screening predictions into conclusive experimental evidence. Furthermore, DR-specific phenotypic assays should be systematically supplemented, such as Evans blue leakage detection, TUNEL apoptosis staining, GFAP immunohistochemistry, and ultrastructural observation by electron microscopy, to improve the therapeutic efficacy evaluation system. Ultimately, multi-omics validation using clinical samples will promote the clinical translation of DHYZ and provide novel theoretical bases and intervention strategies for the precise treatment of DR.

## 5 Conclusion

In this study, we employed an integrated approach combining network pharmacology, metabolomics, and microbiome data to systematically explore the potential biological mechanisms of DHYZ in the treatment of DR. This strategy identified key target genes, critical metabolites, and key microbial taxa, providing new insights into the pathogenesis and treatment of DR. However, a limitation of this study is its reliance on bioinformatic analyses; thus, the findings await further experimental validation in subsequent research.

## Data Availability

The original contributions presented in the study are included in the article/Supplementary material, further inquiries can be directed to the corresponding author.
