# Supplementary material for: Anti-diabetic retinopathy molecular mechanism of Dihuang Yinzi: insights from network pharmacology, metabolomics, and microbiome analysis
Source: Front Med (Lausanne). 2026 May 15;13:1793936. doi: 10.3389/fmed.2026.1793936 (PMC13219277; doi:10.3389/fmed.2026.1793936)
Supplement: Supplementary file 1 [file Table_1.docx]

Supplementary Table

Corresponding Sample Identifiers for Metabolomics and Microbiome Sequencing.
